# Long Noncoding RNA SOX2-OT Exacerbates Hypoxia-Induced Cardiomyocytes Injury by Regulating miR-27a-3p/TGF*β*R1 Axis

**DOI:** 10.1155/2020/2016259

**Published:** 2020-05-06

**Authors:** Guang Yang, Chunsheng Lin

**Affiliations:** ^1^Department of Cardiology, Shaanxi Provincial People's Hospital, Xi'an 710068, Shaanxi, China; ^2^Department of Medical Service, Tianjin Union Medical Center, Tianjin 300121, China

## Abstract

**Background:**

Myocardial infarction (MI) was a severe cardiovascular disease resulted from acute, persistent hypoxia, or ischemia condition. Additionally, MI generally led to heart failure, even sudden death. A multitude of research studies proposed that long noncoding RNAs (lncRNAs) frequently participated in the regulation of heart diseases. The specific function and molecular mechanism of SOX2-OT in MI remained unclear. *Aim of the Study*. The current research was aimed to explore the role of SOX2-OT in MI.

**Methods:**

Bioinformatics analysis (DIANA tools and Targetscan) and a wide range of experiments (CCK-8, flow cytometry, RT-qPCR, luciferase reporter, RIP, caspase-3 activity, trans-well, and western blot assays) were adopted to investigate the function and mechanism of SOX2-OT.

**Results:**

We discovered that hypoxia treatment decreased cell viability but increased cell apoptosis. Besides, lncRNA SOX2-OT expression was upregulated in hypoxic HCMs. Hereafter, we confirmed that SOX2-OT could negatively regulate miR-27a-3p levels by directly binding with miR-27a-3p, and miR-27a-3p also could negatively regulate SOX2-OT levels. Furthermore, knockdown of SOX2-OT promoted cell proliferation, migration, and invasion, but limited cell apoptosis. However, these effects were reversed by anti-miR-27a-5p. Besides, we verified that miR-27a-3p binding with the 3′UTR of TGFBR1 and SOX2-OT regulated TGF*β*R1 level by collaborating with miR-27a-3p in HCMs. Eventually, rescue assays validated that the influence of SOX2-OT silence or miR-27a-3p overexpression on cellular processes in cardiomyocytes injury was counteracted by TGFBR1 overexpression.

**Conclusions:**

Long noncoding RNA SOX2-OT exacerbated hypoxia-induced cardiomyocytes injury by regulating miR-27a-3p/TGF*β*R1 axis, which may provide a novel insight for heart failure treatment.

## 1. Introduction

Myocardial infarction (MI) referred to the necrosis of myocardium caused by severe persistent hypoxia or ischemia due to coronary occlusion and interruption of blood flow [1, 2]. MI brought numerous damages to the heart muscle and has become the leading cause of morbidity and mortality among diseases worldwide [3, 4]. Several risk factors such as excessive labor, irregular diet, smoking, and drinking were reported to be closely associated with the initiation or progression of MI [5, 6]. Although the overall mortality of MI patients was obviously reduced by coronary reperfusion, the additional reperfusion injuries such as cardiomyocyte dysfunction and death were frequently reported [7, 8]. Therefore, better understanding and further exploration of molecular mechanisms in MI are urgently needed.

The long noncoding RNAs (lncRNAs) belonged to a category transcript with no less than 200 nucleotides in length and without protein-coding capacity [9, 10]. LncRNAs were proved to take part in the regulation of several biological processes such as cell proliferation, apoptosis, migration, and invasion in a variety of diseases or tumors [11, 12]. Long noncoding RNA HOTAIR was reported to accelerate the proliferation, migration, and invasion of head and neck squamous cell carcinoma (HNSCC) cells by sponging microRNA-206 and targeting STC2 [13]. Additionally, long noncoding RNA ZFAS1 facilitated chondrocytes proliferation, migration, and apoptosis of in osteoarthritis [14]. In MI, silence of lncRNA XIST inhibited myocardial cell apoptosis through regulating miR-449 in the MI rat model [15]. Recently, lncRNA SOX2 overlapping transcript (SOX2-OT) was proposed to promote a series of tumors progression such as esophageal squamous cell carcinoma, lung squamous cell carcinoma, and cholangiocarcinoma [16–18]. Although SOX2-OT was identified to be upregulated in ischemic heart failure [19], the specific function and regulator mechanism of SOX2-OT remains elusive.

MicroRNAs (miRNAs) were another subgroup of noncoding RNAs with about 22 nucleotides in length [20]. MiRNAs were shown to bind to the 3′ untranslated region (3′-UTR) of target gene to modulate its level and thereby regulate cell proliferation, differentiation, and apoptosis [21]. Numerous miRNAs were proposed to be implicated in the development of MI. As examples, miR-208, miR-494, miR-499, and miR-1303 played key roles in early diagnosis of MI [22]. miR-130 exacerbated myocardial injury caused by acute myocardial infarction via targeting PPAR-*γ* [23]. Recently, the downregulation of miR-27a-3p suppressed inflammatory response and apoptosis of hippocampal neuronal cell in epilepsy via increasing mitogen-activated protein kinase 4 (MAP2K4) [24]. In addition, miR-27a-3p interacted with hsa_circ_0026480 and hsa_circ_0046159 to modulate chronic thromboembolic pulmonary hypertension [25]. Nevertheless, the biological role of miR-27a-3p was unclear in MI.

In conclusion, the present project was aimed to explore the function and mechanism of SOX2-OT in MI. We found out that SOX2-OT exacerbated hypoxia-induced cardiomyocytes injury by promoting proliferation, migration, and invasion but inhibiting apoptosis via miR-27a-3p/TGF*β*R1 axis, which may offer a novel and possible tactic for MI treatment.

## 2. Materials and Methods

### 2.1. Cell and Cell Treatment

Human cardiomyocyte primary cells (HCM) were obtained from Cellprogen (Torrance, USA) and cultivated in human cardiomyocytes primary cell culture media (Cellprogen) in a humid incubator at 37°C with 5% CO_2_. HCMs were incubated in an incubator with 1% O_2_, 5% CO_2_, and 94% N_2_ for different times (12, 24, and 48 h) to induce hypoxia.

### 2.2. Cell Transfection

For cell transfection, miR-27a-3p mimic (miR-27a-3p) and inhibitor (anti-miR-27a-3p) were used to overexpress and knockdown miR-27a-3p with negative control (miR-NC). Short hairpin RNA (shRNA) targeting SOX2-OT (sh-SOX2-OT) with control (NC) was used to inhibit SOX2-OT levels. The full length of SOX2-OT or TGFBR1 was subcloned into pcDNA3.1 vector to overexpress SOX2-OT or TGFBR1 with empty pcDNA3.1 serving as control. All these vectors were purchased from transfected into cells using Lipofectamine 2000 (Invitrogen, USA) following manufacture's protocol.

### 2.3. Real-Time Reverse-Transcription Polymerase Chain Reaction (RT-qPCR)

The extraction of total RNA from HCMs was performed using Trizol reagent (Thermo Fisher, USA) under the manufacturer's instructions. Then, SuperScript® III First-Strand Synthesis System (Thermo Fisher) RNA was used for reverse-transcription. Real-time PCR was operated by QuantiTect SYBR Green PCR Kit (Qiagen, Germantown, USA) on a QuantStudio 3 Real-Time PCR System (Thermo Fisher). The primers would be provided under requirement. GAPDH served as an internal control for lncRNA and mRNA. U6 served as internal control for miRNA. The relative RNA levels were analyzed using the 2^−ΔΔCt^ method.

### 2.4. Luciferase Reporter Assay

About 5 × 10^4^ HCMs were cotransfected with miR-27a-3p mimics, anti-miR-27a-3p or miR-NC and wild-type or mutant SOX2-OT (or 3′UTR of TGFBR1) pGLO plasmids using Lipofectamine 2000 (Invitrogen). All the plasmids were obtained from Genepharma (Shanghai, China). Luciferase activity was detected by a Dual-Luciferase reporter assay system (Promega, USA) after transfection for 48 hours.

### 2.5. RNA Immunoprecipitation (RIP) Assay

Magna RNA immunoprecipitation (RIP) kit (Millipore, Billerica, USA) was adopted in RIP assay. Magnetic beads containing Ago2 or IgG (negative control) antibodies were added into HCM cells lysate which was preserved in RIP buffer before. The relative level of SOX2-OT and miR-27a-3p were detected by RT-qPCR assay. Input served as control.

### 2.6. Cell Viability

Cell Counting Kit-8 (CCK-8; KeyGEN, Nanjing, China) was utilized to test cell viability. In short, 5 × 10^3^ HCMs induced by hypoxia for different times (0, 12, 24, and 48) were seeded into each well in 96-well plates. HCMs were incubated with 10 *μ*L CCK8 solution. The absorbance was measured with 450 nm wavelength in a microplate reader (168-1000 Model 680, Bio-Rad).

### 2.7. Apoptosis Assay by Flow Cytometry

Apoptosis was analyzed through dual staining with the Annexin V-FITC/staining kit (ThermoFisher). According to the manufacturer's instructions, Annexin V-FITC and PI were added to the cellular suspension for staining, FACSCalibur flow cytometer in CellQuest 3.0.1 software (BD Biosciences, USA) was used to analyze HCMs. Percentages of apoptosis HCMs were detected by dual-color analysis.

### 2.8. Caspase-3 Activity Assay

The caspase-3 assay kit (colorimetric) (Abcam, Cambridge, USA) was applied to measure caspase-3 activity in line with manufacturer's protocol. In brief, transfected HCMs were lysed in lysis buffer and then added to 96-well plate after cultivation for 48 hours. Subsequently, caspase-3 catalytic substrate DEVD-pNA, 2× reaction buffer, and DTT were cocultivated with HCMs in each well at 37°C for 2 h. Finally, the measurement of OD value at 405 nm was performed in a microplate reader.

### 2.9. Western Blot

Total protein was extracted from heart tissues and cells by using RIPA lysis buffer (Santa Cruz Biotechnology, USA). The proteins were isolated by SDS-PAGE and then moved onto PVDF membranes (Millipore, MA). Subsequently, Tris-buffered saline 0.01% Tween 20 (Santa Cruz Biotechnology) with 5% defatted milk was used to block the membranes for one hour at room temperature. Next, primary antibodies including anti-TGFBR1 (ab31013, Abcam) and anti-GAPDH (ab181602, Abcam) were cultured with membranes at 4°C overnight. After cultivation with the secondary antibody for 1 h, the blots were visualized with a Clarity™ Western ECL Blotting Substrates (Bio-Rad) under manufacturer's instruction, and protein levels were quantified by using ImageJ software. GAPDH functioned as an internal control.

### 2.10. Trans-Well Assay

The trans-well assays were operated in 24-well trans-well chambers (8 *μ*m; Corning, Shanghai, China). The upper chambers with (or without) 1 mg/mL Matrigel were used to detect cell invasion (or migration). 10, 000 HCMs in medium were seeded into the upper chambers, whilst medium supplemented with 10% FBS was added to the bottom chambers. 24 hours later, the migrated or invaded HCMs were immobilized in 75% methanol and stained with 0.1% crystal violet. The number of migrated and invaded cells in each field was counted under a light microscope.

### 2.11. Statistical Analysis

All data were displayed as means ± SD. Statistical analysis was progressed using the SPSS (Chicago, IL, USA) and GraphPad Prism 5 software (San Diego, CA). Experiments were operated for three times. Significance of the variance between 2 or more groups was evaluated through Student's *t*-test or ANOVA. *P* < 0.05 was regarded as statistical significance.

## 3. Result

### 3.1. SOX2-OT Was Upregulated in HCM Cells Induced by Hypoxia

**Figure 1:**
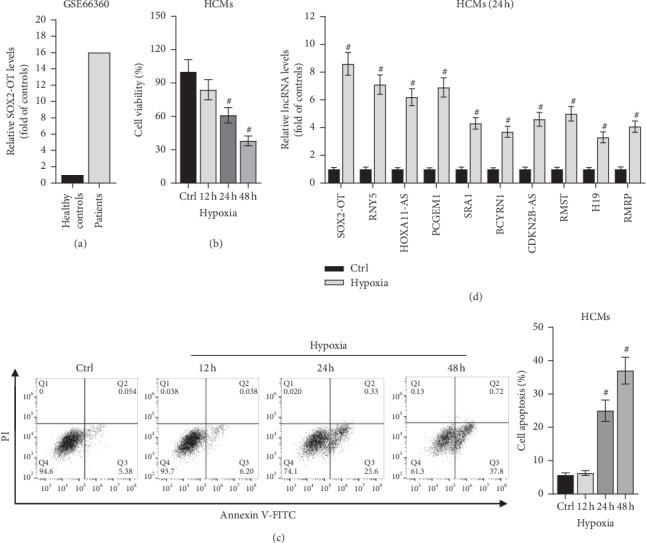
SOX2-OT is upregulated in HCMs treated by hypoxia. (a) Data from GSE66360 showed an upregulation of SOX2-OT in patients' blood. (b) CCK-8 assay was used to measure cell viability. #*P* < 0.01 vs. control. (c) Flow cytometry was utilized to assess cell apoptosis. #*P* < 0.01 vs. control. (d) RT-qPCR was performed to detect lncRNAs (SOX2-OT, RNY5, HOX11-AS, PCGEM1, SRA1, BCYRN1, CDKN2B-AS, RMST, H19, and RMRP) levels. #*P* < 0.01 vs. control.

Previous study has identified several upregulated lncRNAs including SOX2-OT in ischemic heart failure tissues. Additionally, data from GSE66360 (https://www.ncbi.nlm.nih.gov) depicted that SOX2-OT was also upregulated in ischemic cardiomyopathy or myocardial infarction patients' peripheral blood compared with healthy peripheral blood (Figure 1(a)). To further explore the function of SOX2-OT, HCMs were treated with hypoxia for different time to construct the myocardial injury cell model. As shown in Figures 1(b) and 1(c), after treated with hypoxia for 24 or 48 hours, HCMs viability was declined, but the apoptosis rate of HCMs was increased. According to the result of RT-qPCR, SOX-OT displayed the most significant upregulation among these lncRNAs compared with the control group (Figure 1(d)). To sum up, SOX2-OT was upregulated in HCM cells induced by hypoxia.

### 3.2. SOX2-OT Directly Interacted with miR-27a-3p in HCM Cells

**Figure 2:**
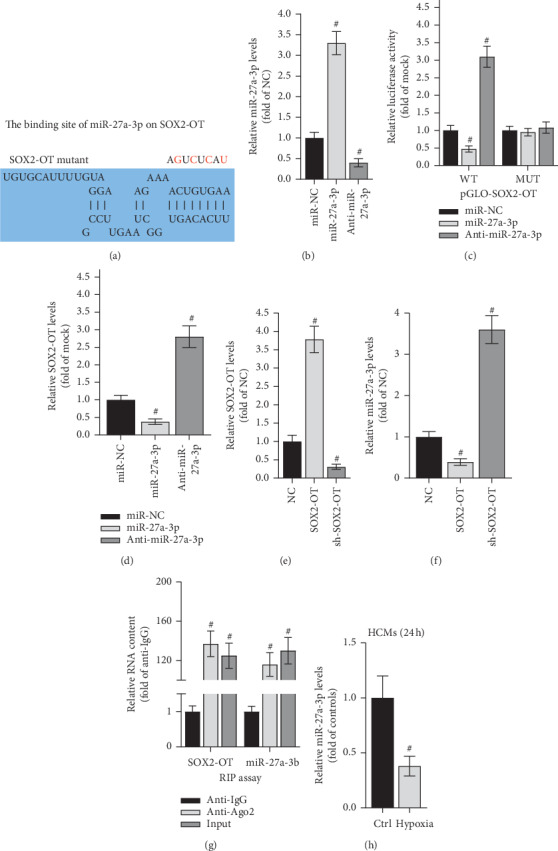
SOX2-OT directly interacts with miR-27a-3p in hypoxic HCMs. (a) The binding site of miR-27a-3p on SOX2-OT was predicted by DIANA tools. (b, e) The overexpression and knockdown efficacy of miR-27a-3p or SOX2-OT was conducted in RT-qPCR. #*P* < 0.01 vs. miR-NC. (c) The binding capacity between SOX2-OT and miR-27a-3p was evaluated by using 4 mutations of SOX2-OT in luciferase reporter assay. #*P* < 0.01 vs. miR-NC. (d, f) RT-qPCR validated that SOX2-OT and miR-27a-3p negatively regulated each other. #*P* < 0.01 vs. miR-NC or NC. (g) RIP assay was adopted to confirm the combination between SOX2-OT and miR-27a-3p. #*P* < 0.01 vs. IgG. (h) The level of miR-27a-3p in HCM cells was measured by RT-qPCR. #*P* < 0.01 vs. control.

In mechanism, SOX2-OT has been widely reported to act as a ceRNA to regulate the level of the downstream target gene. Hence, we anticipated that SOX2-OT also functioned in this way in HCMs. DIANA tools (http://carolina.imis.athena-innovation.gr/diana_tools) were adopted to predict the binding site of miR-27a-3p on SOX2-OT (Figure 2(a)). The result of RT-qPCR validated that the expression of miR-27a-3p was dramatically overexpressed by miR-27a-3p mimics and knocked down by anti-miR-27a-3p in HCMs, respectively (Figure 2(b)). Luciferase reporter assay using 4 mutations of SOX2-OT suggested that the luciferase activity of pGLO-SOX2-OT-WT was, respectively, lowered by miR-27a-3p mimics but raised by anti-miR-27a-3p, whilst no such alteration has been noticed in pGLO-SOX2-OT-MUT (Figure 2(c)). Likewise, SOX2-OT level was also lessened by miR-27a-3p overexpression but elevated by miR-27a-3p knockdown (Figure 2(d)). Hereafter, the level of SOX2-OT was increased (or decreased) by transfecting pcDNA3.1/SOX2-OT (or anti-SOX2-OT) into HCMs (Figure 2(e)). RT-qPCR assay disclosed that miR-27a-3p expression was downregulated by SOX2-OT overexpression but upregulated by SOX2-OT deficiency (Figure 2(f)). At last, RIP assay illustrated that both SOX2-OT and miR-27a-3p were enriched in the anti-Ago2 group but not the anti-IgG group (Figure 2(f)). In conclusion, SOX2-OT directly interacted with miR-27a-3p in HCM cells.

### 3.3. SOX2-OT Cooperated with miR-27a-3p to Modulate Cellular Processes in HCM Cells

**Figure 3:**
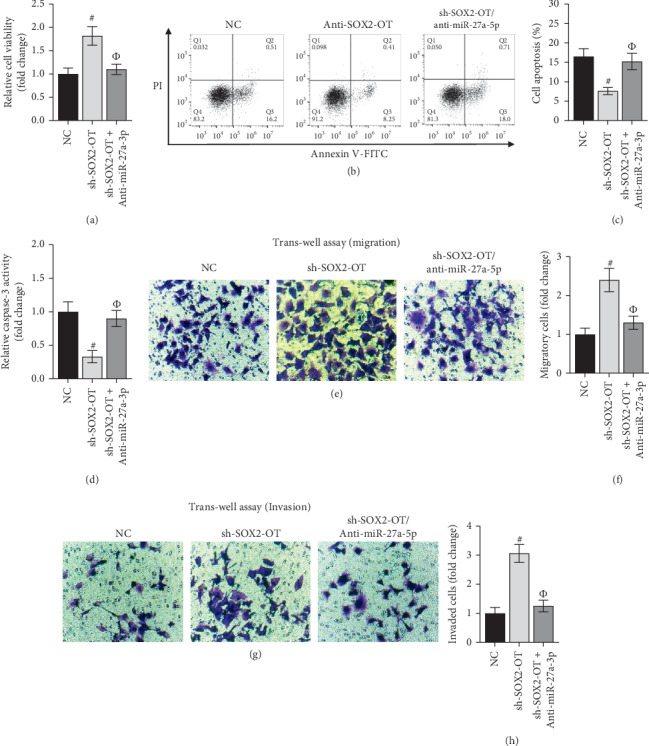
SOX2-OT cooperates with miR-27a-3p to modulate cellular processes of hypoxic HCMs. (a–e) Proliferation, apoptosis, caspase-3 activity, migration, and invasion of HCMs were determined by CCK-8, flow cytometry, caspase-3 activity, and trans-well assays separately. #*P* < 0.01 vs. NC and Φ*P* < 0.01 vs. sh-SOX2-OT.

To explore the specific function of SOX2-OT and miR-27a-3p on cellular processes in HCMs, a wide range of experiments were carried out. To begin with, cell viability was improved by SOX2-OT inhibition and the promoting effect of SOX2-OT silencing were offset by the knockdown of miR-27a-3p (Figure 3(a)). On the contrary, the depletion of miR-27a-3p neutralized the suppressive effect of SOX2-OT attenuation on cell apoptosis and caspase-3 activity (Figures 3(b) and 3(d)). At last, trans-well assay revealed that sh-SOX2-OT-mediated the elevation of migrated and invaded cells was reversed by cotransfection of sh-SOX2-OT and anti-miR-27a-3p (Figures 3(e)–3(h)). Taken together, SOX2-OT cooperated with miR-27a-3p to modulate cellular processes in HCM cells.

### 3.4. SOX2-OT Regulated TGF*β*R1 Level by Collaborating with miR-27a-3p in HCM

**Figure 4:**
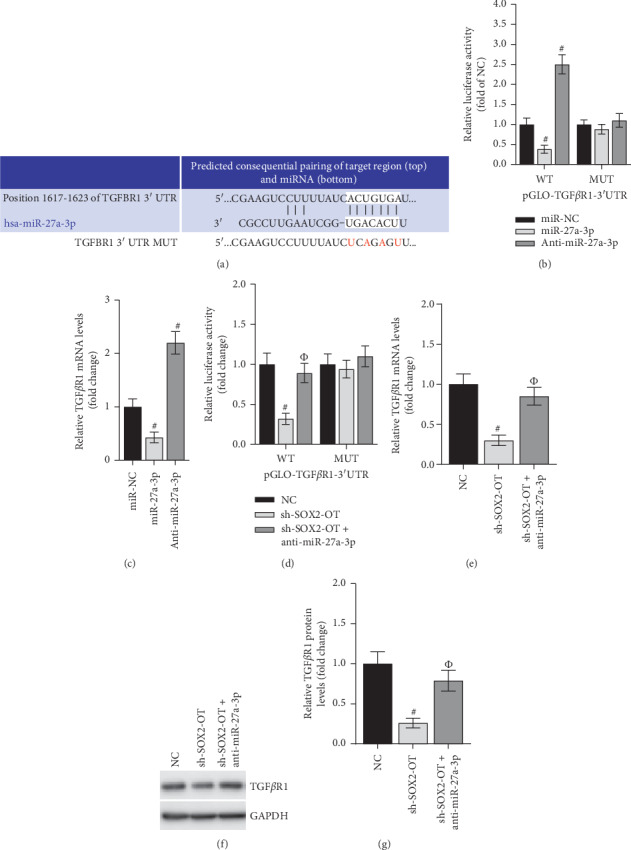
SOX2-OT regulates TGF*β*R1 level by collaborating with miR-27a-3p in hypoxic HCMs. (a) The binding sequences between miR-27a-3p and TGFBR1 were hypothesized by Targetscan. (b, d) The luciferase activity of wild-type or mutant pGLO-TGFBR1-3′UTR was measured by luciferase reporter assay in HCMs. #*P* < 0.01 vs. miR-NC or NC. Φ*P* < 0.01 vs. sh-SOX2-OT. (c, e) The mRNA level of TGFBR1 was monitored by RT-qPCR assay. #*P* < 0.01 vs. miR-NC or NC. Φ*P* < 0.01 vs. sh-SOX2-OT. (f, g) The detection of TGFBR1 protein level was carried out in western blot assay. #*P* < 0.01 vs. Mock/NC and Φ*P* < 0.01 vs. Double KD.

TGFBR1 has been reported to regulate cardiovascular disease. Bioinformatics analysis (Targetscan; http://www.targetscan.org) was used to predict the binding sequences between miR-27a-3p and TGFBR1 3′UTR (Figure 4(a)). Then, luciferase reporter assay demonstrated that the luciferase activity of pGLO-TGFBR1-3′UTR-WT was lessened by miR-27a-3p mimic but strengthened by miR-27a-3p deficiency (Figure 4(b)). RT-qPCR assay delineated that the TGFBR1 mRNA level was downregulated by transfection of miR-27a-3p mimics but upregulated by transfection of anti-miR-27a-3p (Figure 4(c)). Hereafter, we attended to validate that TGFBR1 was regulated by SOX2-OT/miR-27a-3p axis. According to luciferase reporter assay, inhibition of SOX2-OT limited the luciferase activity of wild-type pGLO-TGFBR1-3′UTR, while the alteration of luciferase activity was counteracted by the cotransfection of sh-SOX2-OT and anti-miR-27a-3p (Figure 4(d)). Similarly, the mRNA and protein levels of TGFBR1 were reduced by SOX2-OT suppression and then recovered by the knockdown of miR-27a-3p (Figures 4(e)–4(g)). Taken together, SOX2-OT regulated TGF*β*R1 level by collaborating with miR-27a-3p in HCM.

### 3.5. MiR-27a-3p Inhibited Heart Failure Development by Modulating TGF*β*R1 in HCM Cells

**Figure 5:**
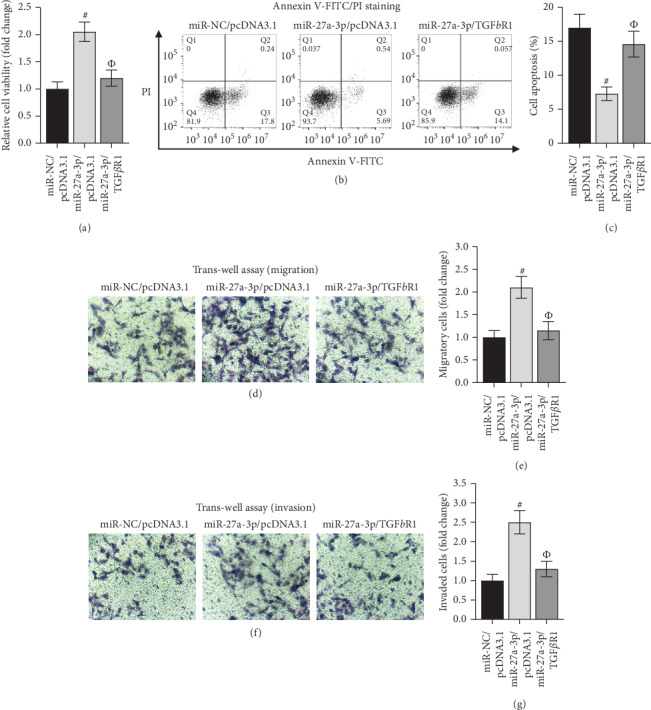
MiR-27a-3p inhibits heart failure development by modulating TGF*β*R1 in HCM cells. (a–g) CCK-8, flow cytometry, caspase-3 activity, and trans-well assays were separately implemented to evaluate proliferation, apoptosis, migration, and invasion of HCMs in miR-NC/pcDNA3.1, miR-27a-3p/pcDNA3.1, and miR-27a-3p/TGF*β*R1 groups. #*P* < 0.01 vs. miR-NC/pcDNA3.1 and Φ*P* < 0.01 vs. miR-27a-3p/pcDNA3.1.

Rescue assays were conducted to confirm the miR-27a-3p/TGF*β*R1 axis in HCM cells. At the beginning, TGFBR1 overexpression inhibited cell viability and reversed the promotive impact of miR-27a-3p mimics on cell viability (Figure 5(a)). Conversely, TGFBR1 overexpression retarded the inhibitive function of miR-27a-3p mimics on cell apoptosis (Figures 5(b) and 5(c)). The miR-27a-3p mimics induced the augmentation of migrated and invaded cells was repressed by ectopic TFGBR1, which also reduced the number of migrated and invaded cells compared with the mock/pcDNA3.1 group (Figures 5(d)–5(g)). In conclusion, miR-27a-3p inhibited heart failure development by modulating TGF*β*R1 in HCM cells.

### 3.6. SOX2-OT Facilitated HCM Cells Injury via TGF*β*R1

**Figure 6:**
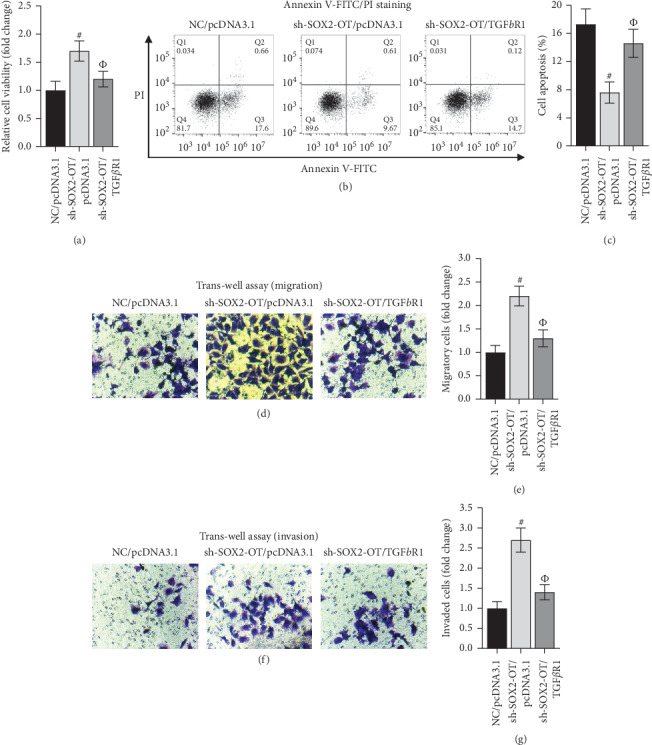
SOX2-OT facilitates HCM cell injury via TGF*β*R1. (a–g) Proliferation, apoptosis, migration, and invasion of HCMs transfected with NC/pcDNA3.1, sh-SOX2-OT/TGF*β*R1, or sh-SOX2-OT/pcDNA3.1 were assessed by CCK-8, flow cytometry, caspase-3 activity, and trans-well assays. #*P* < 0.01 vs. miR-NC/pcDNA3.1 and ФP<0.01 vs. sh-SOX2-OT/pcDNA3.1.

To explore the relationship between SOX2-OT and TGFBR1, HCM cells transfected with different vectors were used in the following assays. According to Figure 6(a), TGFBR1 overexpression countervailed the motivative impact of SOX2-OT suppression on cell viability. Inversely, sh-SOX2-OT-mediated the drop of cell apoptosis rate was neutralized by TGFBR1 overexpression (Figures 6(b) and 6(c)). On the top of that the restraint of cell migration and invasion caused by SOX2-OT inhibition was also abrogated by TGFBR1 overexpression (Figures 6(d)–6(g)). All the experimental results indicated that SOX2-OT facilitated heart failure by modulating TGF*β*R1 in HCM cells.

## 4. Discussions

In recent years, the occurrence of MI has been increasing in China [26]. About 500,000 individuals have been diagnosed with MI each year at least and approximately 2 million are suffering from MI [27]. The typical symptoms of MI were hypotension, shock, arrhythmia, and heart failure [28]. The prognosis of MI was closely related to the size of infarct size and complication [29]. Hence, figuring out more molecules for prognostic marker is necessary.

Although lncRNAs lacked the ability to encode protein, a multitude of research studies reported that lncRNAs were supposed to regulate the development of diverse diseases by modifying nucleic acids or proteins, sponging microRNAs, activating, or inactivating signal molecules [30]. More importantly, emerging reports proposed a competitive endogenous RNA (ceRNA) pattern that lncRNA could competitively combine with miRNA to release mRNA [31, 32]. For instance, lncRNA Malat1 contributed to berberine-mediated suppression of HMGB1 to promote poststroke inflammation by sponging miR-181c-5p [33]. Long noncoding RNA GAS5 was proved to limit cell proliferation and fibrosis by sponging miR-221 and regulating SIRT1 levels in diabetic nephropathy [34]. Recently, a few lncRNAs including SOX2-OT were reported to be upregulated in MI [19, 35]. Consistent with that, in our study, SOX2-OT presented higher level in HCMs induced by hypoxia relative to the control group. Hereafter, we confirmed that SOX2-OT bound with miR-27a-3p, and SOX2-OT negatively regulated miR-27a-3p levels in HCMs, and vice versa. In function, the effects of SOX2-OT knockdown on cellular processes (proliferation, apoptosis, migration, and invasion) were reversed by transfection of anti-miR-27a-3p.

Transforming growth factor beta receptor 1 (TGFBR1), a key molecule in TGF*β*/smad signaling pathway [36], has been widely reported to modulate cellular processes in diseases [37]. For instance, miR-22 regulated the proliferation and differentiation of C2C12 myoblast by targeting TGFBR1 [38]. MicroRNA-98 hampered collagen production and TGF-*β*1-induced differentiation of cardiac fibroblasts via regulating TGFBR1 [39]. In our research, TGFBR1 was directly targeted by miR-27a-3p. The mRNA and protein level of TGFBR1 was regulated by SOX2-OT/miR-27a-3p axis. Previous study claimed that TGFBR1 exacerbated myocardial cell damage induced by hypoxia via inhibiting NF-κB pathway [40]. Similarly, in our exploration, TGFBR1 facilitated proliferation, migration, invasion, and hindered apoptosis of HCMs to aggravate MI. On the top of that, rescue assays suggested that the TGFBR1 amplification offsets the influences of miR-27a-3p mimics or sh-SOX2-OT on cell proliferation, apoptosis, migration, and invasion.

All in all, we confirmed that SOX2-OT exacerbated hypoxia-induced cardiomyocytes injury by regulating miR-27a-3p/TGF*β*R1 axis for the first time, implying a potential diagnostic or therapeutic target for MI patients. However, this was just the initial investigation of SOX2-OT in MI, and other mechanisms remain to be explored in the future.

## Data Availability

The data that support the findings of this study are available from the corresponding author upon reasonable request.

## References

[1] Bianco J. A., Alpert J. S. (1986). Current and future role of noninvasive studies in acute myocardial ischemia and infarction. *American Journal of Physiologic Imaging*.

[2] Zhang D. Y., Wang B. J., Ma M., Yu K., Zhang Q., Zhang X. W. (2019). MicroRNA-325-3p protects the heart after myocardial infarction by inhibiting RIPK3 and programmed necrosis in mice. *BMC Molecular Biology*.

[3] Chistiakov D. A., Orekhov A. N., Bobryshev Y. V. (2016). Cardiac-specific miRNA in cardiogenesis, heart function, and cardiac pathology (with focus on myocardial infarction). *Journal of Molecular and Cellular Cardiology*.

[4] Bonnefoy É., Kirkorian G. (2011). La mortalité des syndromes coronariens aigus. *Annales de Cardiologie et d’Angéiologie*.

[5] Shido K. (1995). A case-control study of life styles associated with myocardial infarction. *Journal of Medical Science*.

[6] Freiberg M. S., Chang C.-C. H., Kuller L. H. (2013). HIV infection and the risk of acute myocardial infarction. *JAMA Internal Medicine*.

[7] Reed G. W., Rossi J. E., Cannon C. P. (2017). Acute myocardial infarction. *The Lancet*.

[8] Frangogiannis N. G. (2015). Pathophysiology of myocardial infarction. *Comprehensive Physiology*.

[9] Schmitz S. U., Grote P., Herrmann B. G. (2016). Mechanisms of long noncoding RNA function in development and disease. *Cellular and Molecular Life Sciences*.

[10] Chen L.-L. (2016). Linking long noncoding RNA localization and function. *Trends in Biochemical Sciences*.

[11] Martens-Uzunova E. S., Böttcher R., Croce C. M., Jenster G., Visakorpi T., Calin G. A. (2014). Long noncoding RNA in prostate, bladder, and kidney cancer. *European Urology*.

[12] Pant T., Dhanasekaran A., Fang J. (2018). Current status and strategies of long noncoding RNA research for diabetic cardiomyopathy. *BMC Cardiovascular Disorders*.

[13] Li T., Qin Y., Zhen Z. (2019). Long non-coding RNA HOTAIR/microRNA-206 sponge regulates STC2 and further influences cell biological functions in head and neck squamous cell carcinoma. *Cell Proliferation*.

[14] Ye D., Jian W., Feng J., Liao X. (2018). Role of long noncoding RNA ZFAS1 in proliferation, apoptosis and migration of chondrocytes in osteoarthritis. *Biomedicine & Pharmacotherapy*.

[15] Zhang M., Liu H. Y., Han Y. L. (2019). Silence of lncRNA XIST represses myocardial cell apoptosis in rats with acute myocardial infarction through regulating miR-449. *European Review for Medical and Pharmacological Sciences*.

[16] Tian W., Jiang C., Huang Z., Xu D., Zheng S. (2019). Comprehensive analysis of dysregulated lncRNAs, miRNAs and mRNAs with associated ceRNA network in esophageal squamous cell carcinoma. *Gene*.

[17] Teng Y., Kang H., Chu Y. (2019). Identification of an exosomal long noncoding RNA SOX2-OT in plasma as a promising biomarker for lung squamous cell carcinoma. *Genetic Testing and Molecular Biomarkers*.

[18] Wei C. X., Wong H., Xu F., Liu Z., Ran L., Jiang R. D. (2018). IRF4-induced upregulation of lncRNA SOX2-OT promotes cell proliferation and metastasis in cholangiocarcinoma by regulating SOX2 and PI3K/AKT signaling. *European Review for Medical and Pharmacological Sciences*.

[19] Greco S., Zaccagnini G., Perfetti A. (2016). Long noncoding RNA dysregulation in ischemic heart failure. *Journal of Translational Medicine*.

[20] Bhaskaran M., Mohan M. (2014). MicroRNAs. *Veterinary Pathology*.

[21] Tüfekci K. U., Öner M. G., Meuwissen R. L. J., Genç Ş. (2014). The role of microRNAs in human diseases. *miRNomics: MicroRNA Biology and Computational Analysis*.

[22] Li P., Li S.-Y., Liu M., Ruan J.-W., Wang Z.-D., Xie W.-C. (2019). Value of the expression of miR-208, miR-494, miR-499 and miR-1303 in early diagnosis of acute myocardial infarction. *Life Sciences*.

[23] Chu X., Wang Y., Pang L., Huang J., Sun X., Chen X. (2018). miR-130 aggravates acute myocardial infarction-induced myocardial injury by targeting PPAR-*γ*. *Journal of Cellular Biochemistry*.

[24] Miao R., Gong J., Zhang C. (2019). Hsa_circ_0046159 is involved in the development of chronic thromboembolic pulmonary hypertension. *Journal of Thrombosis and Thrombolysis*.

[25] Lu J., Zhou N., Yang P., Deng L., Liu G. (2019). MicroRNA-27a-3p downregulation inhibits inflammatory response and hippocampal neuronal cell apoptosis by upregulating mitogen-activated protein kinase 4 (MAP2K4) expression in epilepsy: in vivo and in vitro studies. *Medical Science Monitor*.

[26] Jiang Z., Dreyer R. P., Spertus J. A. (2018). Factors associated with return to work after acute myocardial infarction in China. *JAMA Network Open*.

[27] Li W., Li M., Gao C. (2016). Impact of type 2 diabetes mellitus on recurrent myocardial infarction in China. *Diabetes and Vascular Disease Research*.

[28] Lu L., Liu M., Sun R., Zheng Y., Zhang P. (2015). Myocardial infarction: symptoms and treatments. *Cell Biochemistry and Biophysics*.

[29] Fujimi K., Saku K. (2016). [Prognosis of silent myocardial infarction]. *Nihon Rinsho Japanese Journal of Clinical Medicine*.

[30] Akhade V. S., Pal D., Kanduri C. (2017). Long noncoding RNA: genome organization and mechanism of action. *Advances in Experimental Medicine and Biology*.

[31] Rashid F., Shah A., Shan G. (2016). Long non-coding RNAs in the cytoplasm. *Genomics, Proteomics & Bioinformatics*.

[32] Shi X., Sun M., Liu H., Yao Y., Song Y. (2013). Long non-coding RNAs: a new frontier in the study of human diseases. *Cancer Letters*.

[33] Cao D.-w., Liu M.-m., Duan R. (2019). The lncRNA Malat1 functions as a ceRNA to contribute to berberine-mediated inhibition of HMGB1 by sponging miR-181c-5p in poststroke inflammation. *Acta Pharmacologica Sinica*.

[34] Ge X., Xu B., Xu W. (2019). Long noncoding RNA GAS5 inhibits cell proliferation and fibrosis in diabetic nephropathy by sponging miR-221 and modulating SIRT1 expression. *Aging*.

[35] Huang S., Tao W., Guo Z., Cao J., Huang X. (2019). Suppression of long noncoding RNA TTTY15 attenuates hypoxia-induced cardiomyocytes injury by targeting miR-455-5p. *Gene*.

[36] Sniegon I., Priess M., Heger J., Schulz R., Euler G. (2017). Endothelial mesenchymal transition in hypoxic microvascular endothelial cells and paracrine induction of cardiomyocyte apoptosis are mediated via TGFbeta(1)/SMAD signaling. *International Journal of Molecular Sciences*.

[37] Khalil H., Kanisicak O., Prasad V. (2017). Fibroblast-specific TGF-*β*-Smad2/3 signaling underlies cardiac fibrosis. *Journal of Clinical Investigation*.

[38] Wang H., Zhang Q., Wang B. (2018). miR-22 regulates C2C12 myoblast proliferation and differentiation by targeting TGFBR1. *European Journal of Cell Biology*.

[39] Cheng R., Dang R., Zhou Y., Ding M., Hua H. (2017). MicroRNA-98 inhibits TGF-*β*1-induced differentiation and collagen production of cardiac fibroblasts by targeting TGFBR1. *Human Cell*.

[40] Zhang X. L., An B. F., Zhang G. C. (2019). MiR-27 alleviates myocardial cell damage induced by hypoxia/reoxygenation via targeting TGFBR1 and inhibiting NF-*κ*B pathway. *The Kaohsiung Journal of Medical Sciences*.

